# 25(OH)D and 1,25(OH)D vitamin D fails to predict sepsis and mortality in a prospective cohort study

**DOI:** 10.1038/srep40646

**Published:** 2017-01-12

**Authors:** Franz Ratzinger, Helmuth Haslacher, Markus Stadlberger, Ralf L. J. Schmidt, Markus Obermüller, Klaus G. Schmetterer, Thomas Perkmann, Athanasios Makristathis, Rodrig Marculescu, Heinz Burgmann

**Affiliations:** 1 Department of Laboratory Medicine, Division of Medical and Chemical Laboratory Diagnostics, Medical University of Vienna, Vienna, Austria; 2 Department of Medicine I, Division of Infectious Diseases and Tropical Medicine, Medical University of Vienna, Vienna, Austria; 3 Department of Laboratory Medicine, Division of Clinical Microbiology, Medical University of Vienna, Vienna, Austria

## Abstract

The clinical role of vitamin D in sepsis and mortality prediction is controversially discussed. Therefore, we conducted a prospective cohort study on standard care wards, including 461 patients with suspected sepsis fulfilling two or more SIRS criteria. On the first and third day after onset of SIRS symptoms levels of 25(OH)D, 1,25(OH)D and sepsis biomarkers were analysed for their predictive capacity for identifying infection, bacteraemia and an elevated mortality risk. Additionally, several SNPs associated with vitamin D metabolism were evaluated. Bacteraemic patients (28.5%) presented with significantly lower 1,25(OH)D levels than SIRS patients without bacteraemia on the first and third day, while 25(OH)D did not show a predictive capacity. No significant differences of either 1,25(OH)D or 25(OH)D levels were found between SIRS patients with and without infections or between survivors and non-survivors. Sepsis biomarkers, including procalcitonin and CRP, showed a significantly higher discriminatory capacity for these classification tasks. The vitamin D metabolism-related SNPs analysed did not indicate any association with our outcome measures. In conclusion, 1,25(OH)D but not 25(OH)D showed a minor discriminatory value for the prediction of bacteraemia that was inferior to CRP and PCT but both failed to predict sepsis and mortality in a prospective cohort of SIRS patients.

Vitamin D exhibits a plethora of effects on the innate and adaptive immune responses, endothelial function and the mucosal barrier^1^. Vitamin D deficiency, widely found in severely ill patients, is associated with increased susceptibility to infections^2^,^3^,^4^. Available evidence suggests that vitamin D deficiency may be a predictor of sepsis or elevated mortality rate in critically ill patients^5^,^6^,^7^. Moreover, there is a statistically significant seasonal variation in the sepsis incidence and mortality with higher rates in winter^8^, which might be related to seasonal variations of vitamin D levels.

In the body, vitamin D from both endogenous synthesis and dietary sources is first hydroxylated to 25-hydroxyvitamin (25(OH)D) with a long half-life of two to three weeks, consistent with the main form of vitamin D circulation. Its concentration reflects the vitamin D status of the organism. According to the recommendations of the Endocrine Society, 25(OH)D levels below 20 ng/ml (50 nmol/L) are defined as vitamin D deficiency and 25(OH)D levels of 21–29 ng/ml (52.5–72.5 nmol/L) as vitamin D insufficiency^9^. From this pool of relatively inactive 25(OH)D, many cell types, including those of the immune system, have the ability to synthesize the highly active, but short-lived 1,25-dihydroxy-vitamin D (1,25(OH)D) by regulated expression of the CYP27B1 hydroxylase.

Vitamin D presents with complex pro-inflammatory as well as anti-inflammatory effects^10^. The majority of immune cell types, including B-cells, T-cells and antigen presenting cells express the vitamin D receptor (VDR) and are able to synthetize the active 1,25(OH)D, indicating a pivotal paracrine or autocrine function^11^,^12^. The vitamin D/VDR complex directly induces the expression of antimicrobial proteins such as β-defensin or cathelicidin in cells of the innate immune system^13^,^14^. However, preliminary trials with 1,25(OH)D supplementation in critically ill patients could not distinctly increase cathelicidin serum levels^15^. Stimulatory effects of vitamin D on monocytes and macrophages are observed, including an enhancement of phagocytosis and induction of autophagy^16^,^17^. Moreover, 1,25(OH)D shows immunosuppressive potency on lymphocytes, with the inhibition of T-cell proliferation, a shift from a Th1 to a Th2 immune response, increment of regulatory T-cell count and interference of the CD40 interaction of monocytes with CD40L expressing T-cells^18^,^19^. 1,25(OH)D also inhibits B-cell proliferation as well as the formation of plasma cells and induces B-cell apoptosis^20^. Furthermore, 1,25(OH)D impacts metabolic pathways of dendritic cells and impedes their differentiation and maturation^21^. Based on these findings, several clinical studies were conducted to evaluate whether vitamin D might be used as a diagnostic/predictive marker or even as potential therapeutic target during infections. However, most studies were conducted in a retrospective fashion and 25(OH)D levels were measured before the event-related hospital stay^22^,^23^,^24^. Furthermore, many studies were conducted in the ICU (intensive care unit) setting, neglecting standard care wards^25^.

Therefore, we conducted a prospective cohort study of patients with suspected sepsis fulfilling two or more SIRS criteria treated on standard care wards. The predictive capacity of 1,25(OH)D and 25(OH)D was evaluated on the first and third day after onset of SIRS symptoms. Moreover, we hypothesized that low vitamin D levels predicted bacteraemia, infection or mortality occurrence, which might be relevantly influenced by genetic factors. We thus analysed the distribution of various single nucleotide polymorphisms, which are identified as being relevant for the vitamin D metabolism by genome-wide association studies (GWAS^26^,^27^,^28^,^29^,^30^,^31^,^32^).

## Results

**Figure 1:**
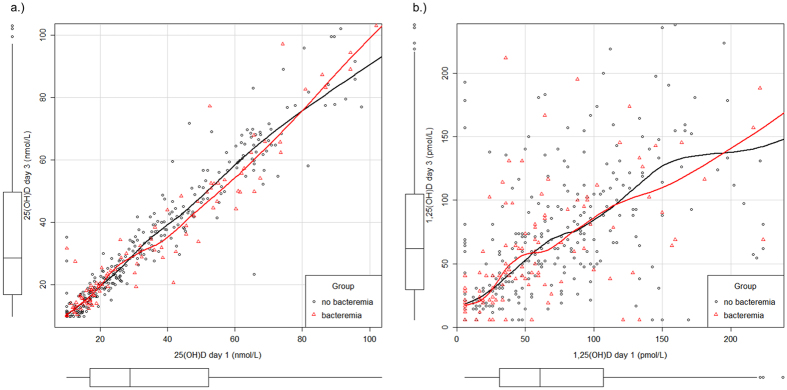
Correlation between 25(OH)D and 1,25(OH)D between the first and the third day within bacteraemic and non-bacteraemic SIRS patients. (**a**) 25(OH)D levels (nmol/L), r_s(25(OH)D)_ = 0.94 (95% CI: 0.96–0.97); (**b**) 1,25(OH)D levels (pmol/L), r_s(1,25(OH)D)_ = 0.60 (95% CI: 0.53–0.66); boxplots show the distribution of data of the corresponding axis, dots outside the box mark extreme values. Red = bacteremia, black = non-bacteremia, lower limit of quantification (LLOQ): 25(OH)D = 10nmol/L, 1,25(OH)D = 5 pmol/L; figure is plotted using car package.

**Figure 2:**
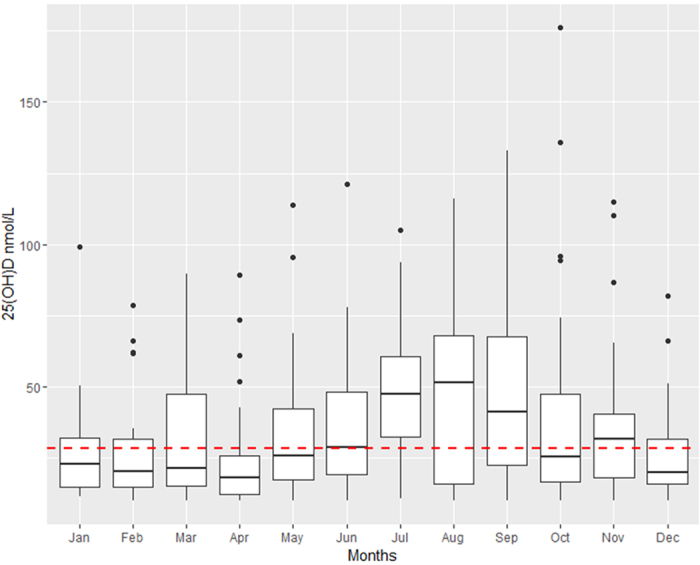
Course of 25(OH)D (nmol/L) during the year. Red dashed line  = overall median 25(OH)D level; boxplots = median with Q1 and Q3, black circles  = outliers, January: 22.8 nmol/L (14.8–32.2), February: 20.4 nmol/L (14.7–31.6), March: 21.3 nmol/L (15.2–47.5), April 18.0 nmol/L (12.1–25.8), May: 26.0 nmol/L (17.5–42.3), June: 28.8 nmol/L (19.3–48.2), July: 47.5 nmol/L (32.6–60.7) August: 51.4 nmol/L (15.9–67.9), September: 41.4 nmol/L (22.4–67.7), October: 25.6 nmol/L (16.8–47.5), November: 31.8 nmol/L (18.0–40.5), December: 20.2 nmol/L (16.0–31.9).

**Figure 3:**
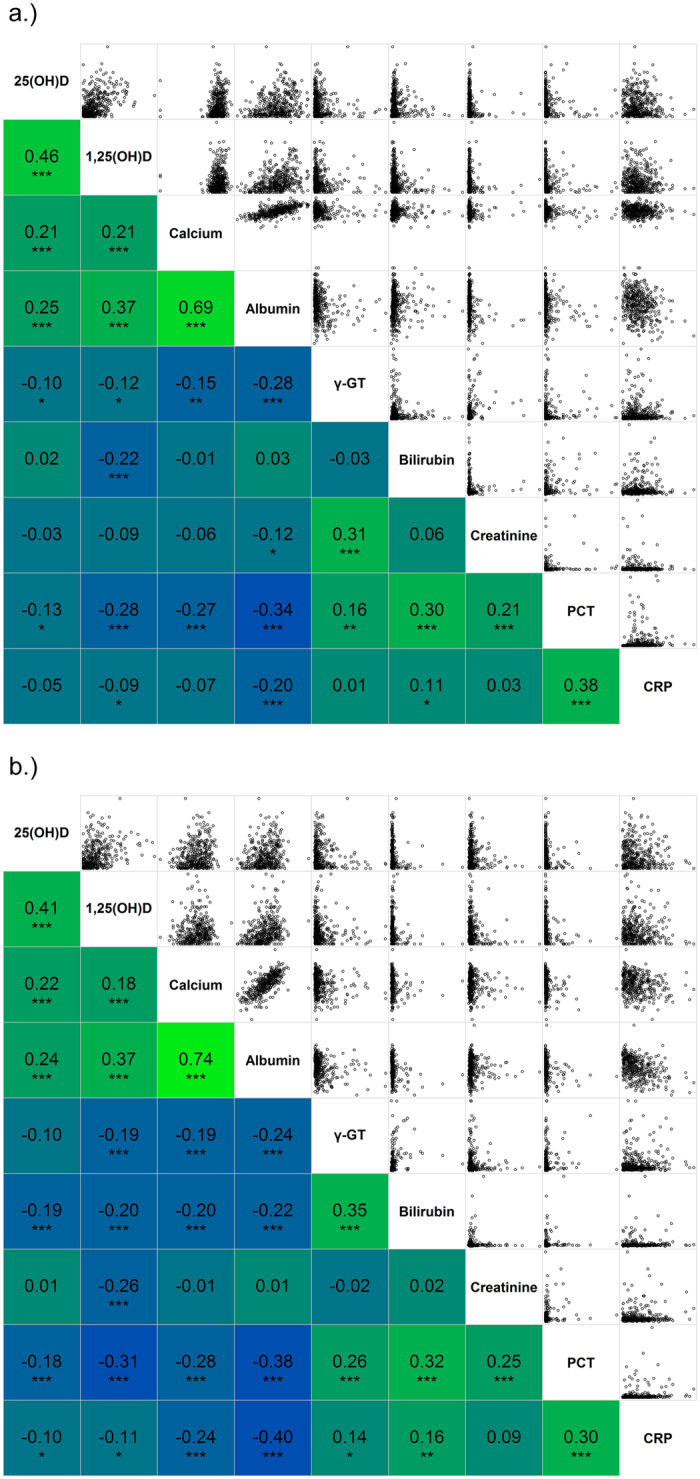
Correlogram of laboratory parameters assessed. (**a**) day 1 after onset of SIRS symptoms; (**b**) day 3 after onset of SIRS symptoms, the head is depicted in the diagonal section; upper right section present the scatter plots of the corresponding r_s_ shown in the lower left section; colour scheme is choose to represent the magnitude and direction of correlation starting with bright blue for r_s_ = −1 to bright green for r_s_ = 1; stars indicate statistical significance of the correlation coefficient as following: *** p < 0.001, **p < 0.01, *p < 0.05; correlograms are drawn with the corrgram package.

A total of 3,370 patients with suspected sepsis were screened. Among these, 2,750 presented with less than two SIRS criteria and 159 patients met at least one exclusion criterion. In total, 461 patients with two or more SIRS criteria were recruited, including 128 patients with bacteraemia (27.8%) and 135 patients suffering from a SIRS-syndrome without any infectious focus (29.3%). Supplementary Table 1 presents the distribution on infection loci according to the ECDC criteria established for point prevalence studies^33^. A median 25(OH)D level of 28.3 nmol/L (16.7–51.8) was found on the first day after onset of SIRS symptoms and 28.7 nmol/L (16.8–49.9) on the third day after onset of SIRS symptoms, with a strong correlation between both time points (r_s_ = 0.96, 95% CI: 0.96–0.97, see Fig. 1). Vitamin D deficiency was seen in 332 patients (73.4%) and a vitamin D insufficiency in 75 patients (16.6%). Since ultra-violet B radiation promotes cutaneous 25(OH)D synthesis^34^, the vitamin was unevenly distributed during the course of the year (p < 0.001), with higher levels in summer months (see Fig. 2). There was a 1,25(OH)D median of 61.8 pmol/L (30.9–107.1) on the first day and 59.5 pmol/L (30.9–107.1) on the third day with a significantly lower correlation (r_s_ = 0.60, 95% CI: 0.53–0.66) between both time points compared to 25(OH)D (p < 0.001). 190 patients (46.6%) had lower 1,25(OH)D levels on the third than on the first day, 40 patients (9.8%) presented with similar levels and 178 patients (43.6%) had higher levels on the third day than on the first. Further, a high prevalence of hypocalcaemia was found. 18% of study patients had a calcium level ≤ 2 mmol/L on the first day (2.17, 2.03–2.28). The correlation of laboratory parameters for the first and third day is presented in Fig. 3.

### Bacteraemia risk prediction

**Figure 4:**
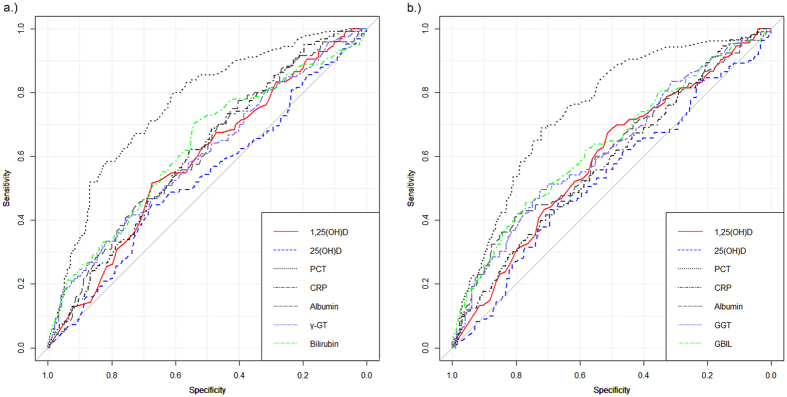
ROC-AUCs of laboratory parameters for identifying bacteraemia in SIRS patients. (**a**) day 1 after onset of SIRS symptoms; ROC-AUCs: 1,25(OH)D: 0.59, 25(OH)D: 0.53, PCT: 0.76, CRP: 0.61, albumin: 0.61, γ-GT: 0.60, bilirubin: 0.63; (**b**) day 3 after onset of SIRS symptoms. ROC-AUCs: 1,25(OH)D: 0.59, 25(OH)D: 0.54, PCT: 0.75, CRP: 0.58, albumin: 0.61, γ-GT: 0.62, bilirubin: 0.63; ROC-curves are plotted with the pROC package^60^.

Bacteraemic patients presented with significantly lower 1,25(OH)D levels than those without bacteraemia (p = 0.004, see Table 1). Hence the predictive value of 1,25(OH)D (day 1) can be expressed as an AUC (area under the curve) of 0.59 (95% CI: 0.53–0.64, see Fig. 4) in receiver operating characteristic plots. When applying the Youden index method, a sensitivity of 0.54 and a specificity of 0.67 was found if 46.4 pmol/l was applied as a cut-off value. No predictive capacity was detected between the temporal development of 1.25(OH)D levels and the occurrence of bacteraemia (ROC-AUC: 0.57, 95% CI: 0.46–0.59). Among those with a decrease of 1,25(OH)D, the proportion of bacteraemic patients was 24.7%, whereas in those with an increase of 1.25(OH)D, 31.5% bacteraemic patients were found (p = 0.403). No significant difference was observed in 25(OH)D levels between bacteraemic and non-bacteraemic patients. Other laboratory parameters, including PCT, CRP or bilirubin, γ-glutamyl transferase or albumin, presented a higher discriminatory capacity than 1,25(OH)D for discriminating between bacteraemic and non-bacteraemic patients.

### Infection risk prediction

No statistically significant difference was found in 1,25(OH)D levels between those with or without infections (see Table 2). Also the 25(OH)D levels between those with and without infection were similarly distributed on the first day and third day after onset of SIRS symptoms. In contrast, CRP and PCT levels on the first and third day after onset were significantly elevated in those SIRS patients with infection compared to those without infection (p_range_ = < 0.001–0.001).

### Mortality risk prediction

Among the study population, 52 patients (11.2%) died during their hospital stay. A statistically insignificant tendency of lower 1,25(OH)D levels was found in non-surviving patients on the first and third day after onset (see Table 3). A similar tendency was observed in 25(OH)D levels on the first and third day after onset of symptoms. In contrast, PCT and CRP were significantly elevated in non-surviving patients when compared to surviving patients (p_range_ = < 0.001–0.001; see Table 3).

### Distribution of SNPs related to the vitamin D metabolism

In the analysis of SNPs evaluated within the study, a significant association to the 25(OH)D levels on the first day was observed for rs10741657 and rs7041 (see Supplementary Table 2). SIRS-patients with rs7041:CA presented with lower levels of 25(OH)D than patients with rs7041:CC (p = 0.006). Similarly, SIRS-patients with an rs10741657:GA had higher 25(OH)D levels than patients with rs10741657:GG (p = 0.027). Further, patients with rs10741657:AA showed lower 1,25(OH)D levels on the third day when compared to those patients with GA (p = 0.007) or GG (p = 0.032). This tendency in the 1,25(OH)D distribution was also found on the first day, but did not yield to statically significance. Furthermore, no statistically significant association was observed between the distribution of SNPs related to the vitamin D metabolism and the occurrence of bacteraemia, infection or mortality (see Supplementary Table 3). A logistic regression model, including clinical data, laboratory parameters and SNPs was not able to improve the predictive accuracies of PCT (data not shown).

## Discussion

The diagnostic or prognostic value of vitamin D for severely ill patients is controversially discussed^25^,^35^,^36^. In the present SIRS-patient cohort recruited in standard care wards, a high prevalence of vitamin D deficiency or insufficiency was found, with less than 10% of patients displaying sufficient 25(OH)D serum levels. No difference in 25(OH)D levels was observed between patients with and without bacteraemia, with and without infections or between surviving and non-surviving patients. Thus the importance of Vitamin D on several immune functions, as shown in a multitude of basic science studies, does not seem to translate into a prognostic or diagnostic value in this SIRS patient cohort. However, due to the plethora of potential vitamin D actions on different cell and tissue types, it is difficult to identify a direct cause-effect relationship to sepsis in our SIRS patient^1^. One might speculate that inflammation regardless of the causative stimulus is associated with low vitamin D levels^1^,^37^. Along those lines, patients with a higher morbidity rate (as in our tertiary SIRS patient cohort) have lower vitamin D levels. Otherwise, a reduced ability to control localized infections might be related to deficient vitamin D levels, subsequently accelerating a systemic host response.

An opposite result regarding the predictive capacity of 25(OH)D for identifying sepsis and elevated mortality risk was found in two meta-analyses, which mainly^24^ or exclusively^25^ included ICU patients. In the most recent meta-analysis, the odd ratio (OR) for developing sepsis ranged between 1.45 (CI: 1.26 to 1.66) and 1.78 (CI: 1.55–2.03^24^). However, between 92.5% to 94% of patients evaluated were retrospectively recruited in the same two teaching hospitals, the Brigham and Women’s Hospital (BWH) and the Massachusetts General Hospital (MGH, both: Boston, Massachusetts, USA). Since the authors of the meta-analysis did not state a strategy to weight these studies involving partly overlapping patient cohorts, the results have to be regarded with caution^5^,^22^. Further, studies conducted at the BWH/MGH exhibited several differences to our study, including the recruitment strategy, patient settings or the time point of 25(OH)D analysis (up to 365 days before suspected sepsis episode), making it difficult to compare the findings. Moreover, due to the long observation period, different methods (radioimmunoassay, chemiluminescence assay, mass spectrometry) for measuring 25(OH)D levels were used, which significantly impacted their results^23^. The work of Braun *et al*., which is comparable to our study in terms of the 25(OH)D measurement time point, showed that ICU patients with 25(OH)D levels below 15 ng/mL had a higher risk of sepsis and 30-day mortality^38^. Using this cut-off value, 61% of our patients would be classified into this high risk group, which had a similar bacteraemia rate or infection rate but elevated mortality rate (13.6% vs. 6.9%, p = 0.031) than the low risk group. However, other parameters were better suited for identifying patients with a higher mortality rate (see Table 3). Within this meta-analysis, only one study with an elevated OR for patients with sepsis was not conducted at BWH/MGH (1.89, 95% CI: 1.09–3.31)^38^. In this retrospective study with community living adults, 211 sepsis patients had lower 25(OH)D levels in median than 1:1 matched controls (61.2 nmol/L; 69.1 nmol/L), which were both in a higher range than our SIRS-patients. Furthermore, the time point of 25(OH)D measurement considerably varied (up to 15 months before the sepsis episode), and sepsis patients presented with a significantly higher rate of comorbidities. Thus, confounding effects of these comorbidities on both serum 25(OH)D levels and the risk of infection cannot be excluded. A second meta-analysis was conducted by de Haan *et al*., evaluating the risk of sepsis, in-hospital mortality and 30-day mortality in ICU treated patients^25^. The risk ratio (RR) for sepsis was 1.46 (CI: 1.27–1.68) for those with less than 50 nmol/L 25(OH)D and the RR for the in-hospital mortality was 1.79 (95% CI: 1.49–2.16). Similar to Upala *et al*.^24^, retrospective studies conducted at BWH/MGH vastly affected their calculations, but a multiple analysis of the same patients seems to be unlikely in their meta-analysis^5^,^39^. Again, only one study not conducted at BWH/MGH with significantly elevated RR for sepsis was included^40^. In this prospective cohort study, including 201 ICU patients, the 25(OH)D insufficient group presented with a significantly higher sepsis rate, but also with a significantly higher rate of organ dysfunctions and ICU mortality. However, a multivariate analysis did not identify 25(OH)D as an independent risk factor for ICU mortality and a multivariate analysis for the sepsis risk was not shown. Further, in three recent studies not included in the meta-analysis, two collected prospectively and one retrospectively, no or only a limited predictive capacity of 25(OH)D for predicting mortality was found^41^,^42^,^43^.

Several publications showed a superiority of the biologically active 1,25(OH)D compared to 25(OH)D for identifying patients with severe infections or a higher risk of mortality^43^,^44^,^45^,^46^,^47^. In a cohort of 3,340 patients after cardiac surgery (5.6% infection rate), those patients with infection showed significantly decreased 1,25(OH)D levels and higher CRP levels, while 25(OH)D was not reduced. Similarly, in patients with infections, the rate of comorbidities and the mortality rate were significantly higher^46^. Accordingly, 1,25(OH)D presented with a predictive capacity for identifying bacteraemia within our SIRS patients cohort. However, other parameters, including PCT, CRP, albumin, bilirubin, or γ-GT, presented a higher discriminatory capacity than 1,25(OH)D. Our data support the hypothesis that reduced levels of 25(OH)D and 1,25(OH)D are rather a marker of systemic inflammation than a marker of severe infection. Jeng *et al*. found a significant difference in 25(OH)D levels between ICU patients and healthy controls; however, a difference between ICU patients with and without sepsis was not found^4^. In this regard, lower 1,25(OH)D levels were also found in patients with hypertension, diabetes, coronary heart disease, heart failure or renal failure^45^. Moreover, creatinine and CRP had an inversely proportional relationship to 1,25(OH)D levels. In our study, a rather high fluctuation was found in the course of 1,25(OH)D concentration between day 1 and day 3 as reflected by the moderate correlation coefficient. However, the direction and amplitude of this fluctuation was not predictive of any analysed clinical outcome.

An association between the occurrence of bacteraemia, infection or mortality and SNPs related to the Vitamin D metabolism was presumed but not verifiable. In contrast to literature^28^,^48^,^49^, SIRS-patients with the rs10741657:AA tended to have lower 25(OH)D and 1,25(OH)D levels than patients with GA or GG. Notably, these previous studies assessed apparently healthy persons or patients without acute illnesses or severe infections^28^,^48^,^49^. Thus, alterations in the vitamin D metabolisms during severe infections might decrease vitamin D levels in patients having rs10741657:AA. The rs10741657 locus is associated with the CYP2R1, which has a vital role in the 25-hydroxylation of vitamin D_2_ and D_3_^50^. The cause for the lower Vitamin D levels in SIRS-patients with rs10741657:AA remains unclear, but these patients also tended to have a higher bacteraemia or mortality rate, which both were not statistically significant (see Supplementary Table 3). Further, computation of haplotypes was conducted but did not lead to an increase of information (data not shown).

Several limitations have to be disclosed in this single centre study. The blood culture analysis was the primary screening criterion requested by the physician in charge. Thus, an observational bias cannot be excluded. Further, specimens for biomarker measurement (day 1) were obtained within 18 hours of the blood culture request. Especially in case of 1,25(OH)D, with an estimated half-life of 4–6 hours^51^, a time-dependent variation in the expression might alter its predictive capacity. However, in the study conducted by Ngyuen *et al*., even a slight decrease in 1,25(OH)D levels was found after 24 hours of ICU stay compared to the initial levels^44^, indicating that the time delay in our study is of minor impact for its predictive capacity.

In conclusion, 25(OH)D had no predictive capacity for identifying infection, bacteraemia or elevated mortality risk in our SIRS patient cohort, while lower 1,25(OH)D levels were found in bacteraemic patients. CRP or PCT are better suited for predictive purposes.

## Materials and Methods

### Study design

The prospective cohort study was approved by the ethics committee of the Medical University of Vienna (EC-No. 518/2011) and was performed in accordance with the Declaration of Helsinki 1964 (including current revisions) and the Good Clinical Practice guidelines of the European Commission. All patients gave written informed consent prior to participation. Between July 2011 and September 2012, patients with clinically suspected sepsis were evaluated on 14 medical and 13 surgical standard care wards of Vienna General Hospital (latitude 48°N), Austria. As described elsewhere^52^,^53^,^54^,^55^, patients for whom the treating physician had requested a blood culture analysis were screened for fulfilment of two or more SIRS criteria, as defined by the ACCP/SCCM conference^56^. Iatrogenic neutropenia related to chemotherapy was not recognized as a valid SIRS criterion. Bacteraemia was defined by the detection of pathogenic bacteria in blood by culture or polymerase chain reaction (PCR) analysis. Blood culture contaminants were specified according to Hall and Lyman^57^. Coagulase-negative staphylococci (CNS) were regarded as invasive pathogens only when detected in two blood specimens taken in separate blood draws. After a patient’s discharge, occurrence of infection was evaluated by applying the definition criteria for hospital-acquired infections of the European Centre of Disease Control (ECDC^33^). These criteria include clinical, laboratory and radiological data. Criteria for classifying SIRS patients without infections could not be found in the literature.

### Data collection

Clinical data was gathered at the time of patients’ enrolment to the study, and was completed after hospital discharge. Blood specimens were cultured in a set of FN Plus (anaerobic) and FA Plus (aerobic) blood culture bottles in the BacT/ALERT 3D automated blood culture system (bioMérieux, Marcy l’Etoile, France). Isolates were identified by matrix-assisted laser desorption ionisation (MALDI) time of flight (TOF) mass spectroscopy (MS) using microflex LT together with the Biotyper database (Bruker Daltonik GmbH, Bremen, Germany), or in the case of *Streptococcus pneumoniae* identification the result was confirmed by optochin disc tests. In some cases, microbial DNA was detected by SeptiFast MGRADE tests (SeptiFast MGRADE tests, Roche Diagnostics GmbH, Mannheim, Germany) as described in^52^,^53^.

Serum 1,25(OH)D levels (reference value: 19.9–79.3 pmol/ml) were assessed using blood archived by the MedUni Wien Biobank (www.biobank.at, date of access: 26/11/2016), a professional biorepository with certified quality management (ISO 9001:2008), and 25(OH)D (reference value: 75–250 nmol/L) by a direct competitive chemiluminescent immunoassay using the DiaSorin Liaison System (DiaSorin, Saluggia, Italy). Further, the following parameters were evaluated: white blood cell counts (WBC; Stromatolyser-4DS; Sysmex, Norderstedt, Germany), CRP (Latex test; Beckman Coulter, Brea, CA, USA), LBP (IMMULITE 2000 Immunoassay System, Siemens Healthcare, Erlangen, Germany), PCT (Hoffmann-La Roche Ltd, Basel, Switzerland) and calcium, albumin, γ-GT, bilirubin, and creatinine (all reagents by Beckman Coulter, Brea, CA, USA).

### Genetic polymorphisms

Seven single nucleotide polymorphisms (SNPs) were analysed at the four confirmed loci previously assessed in GWAS studies^26^,^27^,^28^,^29^,^30^,^31^,^32^. Of those, three SNPs were within or near the GC-gene (vitamin D-binding protein, rs2282679, rs4588, rs7041), two SNPs at the CYP2R1 (ribosomal vitamin D 25-hydroxylase, rs1993116, rs10741657), one SNP at the CYP24A1 (vitamin D-inactivating 24-hydroxylase, rs6013897) and one SNP near the NADSYN1/DHCR7 (7-dehydrocholesterol-reductase, rs12785878) locus. For the analysis, DNA was isolated from peripheral blood by a column-based isolation method (E.Z.N.A.^®^ Blood DNA Mini Kit, Omega Biotek Store Inc., Norcross, USA). The SNPs were assessed using the commercially available TaqMan^®^ SNP Genotyping assays according to the manufacturer’s instructions (Applied Biosystems, Rotkreuz, Switzerland). In detail, 10–50 ng of genomic DNA were amplified in a total volume of 10 μL consisting of 5 μL 2x TaqMan Genotyping Mastermix (Applied Biosystems), 0.5 μL of the respective 20x TaqMan Genotyping Assay and purified water ad 10 μL. PCR conditions were as follows: 10’ at 95 °C (initial denaturation step), followed by 40 cycles alternating at 95 °C for 15” (denaturation) and at 60 °C for 60” (annealing/elongation) on an Applied Biosystems 7900HT fast real-time thermal cycler (Applied Biosystems). Genotypes were determined by post-PCR allelic discrimination using the Applied Biosystems SDS 2.4 software (Applied Biosystems). Within each run, three pre-tested reference DNA samples (Human Reference Panel II, Sigma Aldrich, St. Louis, Missouri) were used as quality controls.

### Statistical analysis

The statistical analysis was performed using R (version 3.3.0, Vienna, Austria^58^). Categorical data are given as counts and percentages and analysed using Fisher’s exact test, numeric data are given as median with Q1 and Q3 and analysed using the Mann-Whitney U test or the Kruskal-Wallis test. For post-hoc testing, Dunn’s test for multiple comparisons with adjusted p-values was applied for controlling the family-wise error rate^59^. Correlation analysis was performed with Spearman’s rank correlation coefficient (r_s_). Multivariable models were computed using a logistic regression model. The predictive capacity of parameters was examined using the area under receiver operating characteristic curve analysis (ROC-AUCs). ROC-AUCs were compared using a distribution-free permutation test^60^. Statistical significance is defined as p-values less than 0.05. Where appropriate, an accumulation of an alpha error related to multiple testing was controlled by the Bonferroni-Holm method.

## Additional Information

**How to cite this article**: Ratzinger, F. *et al*. 25(OH)D and 1,25(OH)D vitamin D fails to predict sepsis and mortality in a prospective cohort study. *Sci. Rep.* **7**, 40646; doi: 10.1038/srep40646 (2017).

**Publisher's note:** Springer Nature remains neutral with regard to jurisdictional claims in published maps and institutional affiliations.

## Supplementary Material

Supplementary Information

## Figures and Tables

**Table 1:** Clinical and laboratory data of bacteraemic and non-bacteraemic SIRS patients.

Parameter	Non- bacteraemic	Bacteraemic	p-value	ROC
n	333 (72.2%)	128 (27.8%)	—	—
Age	56.2 (41.8–68.7)	61.0 (46.3–69.6)	0.570	ns
Female:Male	138:195 (41.4%:58.6%)	59:69 (46.1%:53.9%)	0.400	ns
BMI	24.9 (21.6–29.0)	25.3 (21.3–28.7)	0.723	ns
HBR	99.0 (92.0–108.0)	100.0 (91.0–110.0)	0.462	ns
RR	21.0 (16.0–24.0)	21.0 (17.0–24.0)	0.161	ns
BT	38.5 (38.1–39.0)	38.9 (38.5–39.1)	0.006	ns
WBC (G/L)	11.3 (5.7–15.8)	10.2 (5.9–14.4)	0.329	ns
1,25(OH)D_d1	66.6 (33.3–111.9)	44.0 (24.4–92.2)	0.004*	0.587
1,25(OH)D_d3	67.8 (33.3–111.9)	47.6 (23.8–88.1)	0.004*	0.592
25(OH)D_d1	29.7 (17.0–51.1)	25.9 (16.1–52.5)	0.326	ns
25(OH)D_d3	29.9 (17.5–50.6)	24.8 (15.3–49.7)	0.276	ns
Calcium_d1	2.18 (2.03–2.30)	2.15 (2.04–2.28)	0.227	ns
Calcium_d3	2.18 (2.07–2.30)	2.16 (2.03–2.29)	0.190	ns
Albumin_d1	31.6 (28.2–35.1)	29.9 (25.3–32.9)	<0.001*	0.609
Albumin_d3	32.0 (28.4–36.0)	30.1 (24.9–34.3)	0.001*	0.610
γ-GT_d1	61.5 (29.8–130.5)	92.5 (40.0–247.5)	<0.001*	0.602
γ-GT_d3	64.0 (31.0–134.5)	108.0 (46.5–269.8)	<0.001*	0.618
Bilirubin_d1	0.61 (0.46–0.92)	0.82 (0.58–1.42)	<0.001*	0.627
Bilirubin_d3	0.56 (0.42–0.77)	0.71 (0.48–1.37)	<0.001*	0.633
Crea_d1	0.93 (0.76–1.25)	0.98 (0.79–1.56)	0.193	ns
Crea_d3	0.90 (0.75–1.19)	0.93 (0.74–1.33)	0.655	ns
PCT_d1	0.27 (0.12–0.93)	2.36 (0.45–7.80)	<0.001*	0.760^a^
PCT_d3	0.18 (0.09–0.50)	0.81 (0.31–1.75)	<0.001*	0.747^b^
CRP_d1	13.0 (7.9–20.4)	16.2 (10.7–24.3)	<0.001*	0.608
CRP_d3	7.3 (3.7–13.1)	9.1 (5.1–16.9)	0.012	0.581

BMI = body mass index, HBR = heart beat rate, RR = respiration rate, BT = body temperature, WBC = white blood count,_d1 = day 1,_d3 = day 3, 1,25(OH)D (pmol/ml), 25(OH)D (nmol/L), Ca = calcium (mmol/L), Alb = albumin (g/l), GGT = γ-glutamyl transferase (U/L), Bil = bilirubin (mg/dl), Crea = creatinin (mg/dl), PCT = procalcitonin (ng/ml), CRP = C-reactive protein (mg/dl), ^*^statistical significance after applying the Bonferroni-Holm method, ^a^significantly better than 1,25(OH)D_d1, ^b^significantly better than 1,25(OH)D_d3.

**Table 2:** Clinical and laboratory data of SIRS patients with and without infections.

Parameter	Non-infection	Infection	p-value	ROC
n	135 (29.3%)	326 (70.7%)	—	—
Age	54.0 (38.9–64.2)	60.0 (43.9–70.1)	0.004	0.585
Female:Male	57:78	140:186	0.918	n.s.
BMI	25.2 (21.5–29.4)	25.0 (21.5–28.4)	0.668	n.s.
HBR	100.0 (92.0–108.0)	100.0 (91.3–109.0)	0.652	n.s.
RR	19.5 (15.8–24.0)	21.0 (16.0–24.0)	0.057	n.s.
BT	38.3 (38.0–38.8)	38.5 (38.1–39.0)	0.032	0.563
WBC (G/L)	10.0 (2.5–16.0)	11.3 (6.7–15.3)	0.094	n.s.
1,25(OH)D_d1	66.6 (35.7–111.3)	59.5 (28.6–102.3)	0.198	n.s.
1,25(OH)D_d3	60.7 (36.9–97.6)	59.5 (28.6–107.1)	0.866	n.s.
25(OH)D_d1	28.8 (16.3–48.5)	28.3 (16.9–52.1)	0.929	n.s.
25(OH)D_d3	30.3 (17.0–50.6)	28.5 (16.8–49.8)	0.906	n.s.
Calcium_d1	2.2 (2.0–2.3)	2.2 (2.0–2.3)	0.126	n.s.
Calcium_d3	2.2 (2.1–2.3)	2.2 (2.1–2.3)	0.842	n.s.
Albumin_d1	31.7 (28.2–35.7)	30.9 (26.9–34.4)	0.091	n.s.
Albumin_d3	32.2 (28.6–37.0)	31.2 (27.4–35.0)	0.051	n.s.
γ-GT_d1	67.0 (34.–174.5)	69.5 (31.0–157.5)	0.829	n.s.
γ-GT_d3	64.0 (33.0–144.0)	72.0 (35.5–158.5)	0.372	n.s.
Bilirubin_d1	0.67 (0.49–1.11)	0.67 (0.48–0.99)	0.661	n.s.
Bilirubin_d3	0.65 (0.46–0.99)	0.57 (0.42–0.84)	0.093	n.s.
Crea_d1	0.89	0.95 (0.77–1.30)	0.302	n.s.
Crea_d3	0.91	0.91 (0.75–1.20)	0.898	n.s
PCT_d1	0.27 (0.11–0.70)	0.53 (0.18–2.60)	<0.001*	0.618
PCT_d3	0.18 (0.08–0.45)	0.32 (0.11–1.08)	0.001*	0.610
CRP_d1	10.5 (6.6–16.1)	15.5 (9.8–22.2)	<0.001*	0.637
CRP_d3	5.9 (2.6–10.8)	8.5 (4.9–15.0)	<0.001*	0.627

BMI = body mass index, HBR = heart beat rate, RR = respiration rate, BT = body temperature, WBC = white blood count,_d1 = day 1,_d3 = day 3, 1,25(OH)D (pmol/ml), 25(OH)D (nmol/L), Ca = calcium (mmol/L), Alb = albumin (g/l), GGT = γ-glutamyl transferase (U/L), Bil = bilirubin (mg/dl), Crea = creatinin (mg/dl), PCT = procalcitonin (ng/ml), CRP = C-reactive protein (mg/dl), ^*^statistical significance after applying the Bonferroni-Holm method.

**Table 3:** Clinical and laboratory data of surviving and non-surviving SIRS patients.

Parameter	Survived	Non–survived	p-value	ROC
n	409 (88.7%)	52 (11.3%)	—	—
Age	56.0 (41.9–67.6)	65.3 (58.1–73.1)	0.004	n.s.
Female:Male	169:240	28:24	0.102	n.s.
BMI	25.3 (21.6–29.0)	23.7 (20.2–27.6)	0.668	n.s.
HBR	100 (92.0–108.0)	100.0 (91.8–112.8)	0.645	n.s.
RR	21.0 (16.0–24.0)	21.0 (16.0–24.0)	0.057	n.s.
BT	38.5 (38.1–39.0)	38.5 (37.5–39.6)	0.353	n.s.
WBC (G/L)	11.3 (5.8–15.5)	9.6 (4.6–14.2)	0.095	n.s.
1,25(OH)D_d1	64.3 (33.3–109.5)	40.5 (16.7–73.2)	0.198	n.s.
1,25(OH)D_d3	64.3 (33.3–108.9)	42.8 (17.9–84.5)	0.864	n.s.
25(OH)D_d1	29.8 (16.9–52.8)	24.7 (13.4–36.0)	0.866	n.s.
25(OH)D_d3	29.7 (17.3–50.9)	24.7 (11.2–35.3)	0.868	n.s.
Calcium_d1	2.2 (2.1–2.3)	2.1 (2.0–2.2)	0.126	n.s.
Calcium_d3	2.2 (2.1–2.3)	2.1 (2.0–2.2)	0.842	n.s.
Albumin_d1	31.5 (28.1–35.0)	26.7 (22.5–31.3)	0.092	n.s.
Albumin_d3	32.0 (28.2–36.0)	26.2 (21.8–31.5)	0.052	n.s.
γ-GT_d1	64.0 (30.0–154.5)	102.5 (51.5–227.0)	0.829	n.s.
γ-GT_d3	66.0 (33.0–147.0)	108.0 (50.0–256.0)	0.024	0.605
Bilirubin_d1	0.66 (0.48–1.02)	0.73 (0.49–1.05)	0.661	n.s.
Bilirubin_d3	0.57 (0.42–0.82)	0.84 (0.49–1.24)	0.093	n.s.
Crea_d1	0.93 (0.77–1.29)	0.91 (0.75–1.38)	0.302	n.s.
Crea_d3	0.93 (0.76–1.33)	0.93 (0.76–1.30)	0.898	n.s.
PCT_d1	0.39 (0.15–1.95)	0.53 (0.25–2.39)	<0.001*	0.566
PCT_d3	0.25 (0.10–0.79)	0.63 (0.26–1.59)	0.001*	0.668
CRP_d1	13.4 (8.6–21.0)	15.9 (10.9–24.8)	<0.001*	0.595
CRP_d3	7.5 (4.0–13.8)	10.2 (5.7–16.7)	<0.001*	0.591

BMI = body mass index, HBR = heart beat rate, RR = respiration rate, BT = body temperature, WBC = white blood count,_d1 = day 1,_d3 = day 3, 1,25(OH)D (pmol/ml), 25(OH)D (nmol/L), Ca = calcium (mmol/L), Alb = albumin (g/l), GGT = γ-glutamyl transferase (U/L), Bil = bilirubin (mg/dl), Crea = creatinin (mg/dl), PCT = procalcitonin (ng/ml), CRP = C-reactive protein (mg/dl), ^*^statistical significance after applying the Bonferroni-Holm method.
