# Supplementary material for: 25(OH)D and 1,25(OH)D vitamin D fails to predict sepsis and mortality in a prospective cohort study
Source: Sci Rep. 2017 Jan 12;7:40646. doi: 10.1038/srep40646 (PMC5228346; doi:10.1038/srep40646)
Supplement: Supplementary Information [file srep40646-s1.doc]

25(OH)D and 1,25(OH)D vitamin D fails to predict sepsis and mortality in a prospective cohort study

Franz Ratzinger, Helmuth Haslacher, Markus Stadlberger, Ralf L.J. Schmidt, Markus Obermüller, Klaus G. Schmetterer, Thomas Perkmann, Athanasios Makristathis, Rodrig Marculescu, Heinz Burgmann^*^

*Corresponding author:

Email: [heinz.burgmann@meduniwien.ac.at](mailto:heinz.burgmann@meduniwien.ac.at)

Supplementary Information

**Supplementary table 1: Distribution of infections according to ECDC classification criteria**

**Supplementary table 2: Vitamin D levels in relation to SNPs assessed**

**Supplementary table 3: Distribution of SNP loci assessed regarding bacteraemia, infection and mortality rate**

**Supplementary table 1: Distribution of infections according to ECDC classification criteria** Type = type of infection, modified ECDC class according to

| **Type** | **ECDC class** | **n** | **%** |
| --- | --- | --- | --- |
| **Bloodstream infection^1^** | C-CVC^3^ (n=20), S-DIG^4^ (n=19), S-PUL^5^ (n=13), S-SSI^6^ (n=6), S-SST^7^ (n=6) S-UTI^8^ (n=11), S-OTH^9^ (n=18), S-UO^10^ (n=34) | 128 | 39.3% |
| **Respiratory tract infection^2^** | PN1^11^ (n=3), PN3^12^ (n=6), PN4^13^ (n=4), PN5^14^ (n=64), LRI-Bron^15^(n=3), LRI-Lung^16^(n=2) | 82 | 25.1% |
| **Gastrointestinal system infection^2^** | GI-CDI^17^ (n=4), GI-GE^18^ (n=5), GI-GIT^19^ (n=7), GI-IAB^20^ (n=12), EENT-ORAL^21^ (n=2) | 30 | 9.2% |
| **Urinary tract infection^2^** | UTI-A^22^ (n=20), UTI-B^23^ (n=21) | 41 | 12.6% |
| **Others^2^** | SYS-CESP^24^ (n=7), SYS-DI^25^ (n=7), SSI-S^26^ (n=9), SSI-D^27^ (n=2), SSI-O^28^ (n=1), CVS-Card^29^ (n=2), CVS-Vasc^30^ (n=1), CVS-Endo^31^ (n=2), SST-Skin^32^ (n=4), SST-ST^33^ (n=3), REPR-OREP^34^ (n=2), CRI-CVC^35^(n=3), CNS-IC^36^ (n=1), CNS-SA^37^(n=1), BJ-JNT^38^ (n=1) | 45 | 13.8% |
| **Total** |  | 326 | 100% |

^1^= blood culture positive; ^2^= blood culture negative; ^3^= blood stream infection (BSI), related to central vascular catheter; ^4^= BSI, secondary digestive tract infection; ^5^= BSI, secondary to pulmonary infection; ^6^= BSI, secondary to surgical site infection; ^7^= BSI, secondary to skin and soft tissue infection; ^8^= BSI, secondary to urinary tract infection; ^9^= BSI, secondary to another infection; ^10^= BSI, (confirmed) unknown origin; ^11^= pneumonia, positive quantitative culture from minimally contaminated lower respiratory tract specimen; ^12^= pneumonia, microbiological diagnosis by alternative microbiology methods, ^13^= pneumonia, positive sputum culture or non-quantitative culture from lower respiratory tract specimen; ^14^= pneumonia, clinical signs of pneumonia without positive microbiology; bronchitis, tracheobronchitis, bronchiolitis, tracheitis, without evidence of pneumonia; ^15^= LRI, other infections of the lower respiratory tract, bronchitis, tracheobronchitis, bronchiolitis, tracheitis; ^16^= lower respiratory tract infection, other than pneumonia; ^17^= gastrointestinal system infections (GI) *clostridium difficile* infection; ^18^= GI, gastroenteritis (excluding CDI); ^19^= Gastrointestinal tract (oesophagus, stomach, small and large bowel, and rectum), excluding GE, CDI; ^20^= GI, intra-abdominal infection, not specified elsewhere; ^21^= eye, ear, nose or mouth infection (EENT), oral cavity (mouth, tongue, or gums); ^22^= urinary tract infection (UTI), microbiologically confirmed symptomatic UTI; ^23^= UTI, not microbiologically confirmed symptomatic UTI; ^24^= systemic infections (SYS), clinical sepsis in adults and children; ^25^= SYS, disseminated infection; ^26^= surgical site infection (SSI), superficial incisional; ^27^= surgical site infection (SSI), deep incisional;^28^= surgical site infection, organ/space; ^29^= cardiovascular system infection (CVS), myocarditis or pericarditis; ^30^ = CVS, arterial or venous infection; ^31^= endocarditis, ^32^= skin and soft tissue infections (SST), skin; ^33^= SST, soft tissue (necrotizing fasciitis, infectious gangrene, necrotizing cellulitis, infectious myositis, lymphadenitis, or lymphangitis); ^34^= reproductive tract infections (REPR)-other infections of the male or female reproductive tract (OREP), ^35^= central vascular catheter-related infection (CRI), general CVC-related infection (no positive blood culture); ^36^= central nervous system infection (CNS), intracranial infection; ^37^= CNS-SA: spinal abscess without meningitis; ^38^BJ-JNT= joint or bursa infection.

**Supplementary table 2: Vitamin D levels in relation to SNPs assessed**

| **SNP** | | **n** | **25(OH)D_d1** | | | **1,25(OH)D_d1** | | | **1,25(OH)D_d3** | | |
| --- | --- | --- | --- | --- | --- | --- | --- | --- | --- | --- | --- |
| **rs1993116*** | AA | 114 | 24.8 (16.8–48.4) | 0.050 | n.s. | 53.6 (26.2-102.3) | 0.101 | n.s. | 50.0 (30.9-92.8) | 0.114 | n.s. |
|  | GG | 160 | 26.0 (15.2–45.7) |  | n.s. | 59.5 (32.1-96.4) |  | n.s. | 59.5 (26.8-102.3) |  | n.s. |
|  | GA | 186 | 33.9 (18.5–53.2) |  | n.s. | 71.4 (33.3-119.6) |  | n.s. | 76.2 (30.9-120.3) |  | n.s. |
| **rs10741657** | AA | 81 | 23.1 (16.9–54.2) | 0.049 | 0.149^A^ | 54.7 (30.9-104.7) | 0.192 | n.s. | 47.6 (30.9-80.9) | 0.009 | 0.007^A^ |
|  | GG | 163 | 25.7 (14.9–44.7) |  | 0.333^B^ | 58.3 (29.2-92.8) |  | n.s. | 58.3 (26.2-97.6) |  | 0.142^B^ |
|  | GA | 217 | 32.7 (18.5–52.2) |  | 0.027^C^ | 69.0 (30.9-116.6) |  | n.s. | 76.2 (33.3-126.1) |  | 0.032^C^ |
| **rs7041*** | AA | 75 | 29.1 (17.4–50.5) | 0.016 | 0.148^D^ | 66.6 (31.5-97.6) | 0.432 | n.s. | 64.3 (30.9-115.4) | 0.331 | n.s. |
|  | CA | 228 | 25.8 (15.7–45.3) |  | 0.006^E^ | 59.5 (29.8-107.1) |  | n.s. | 57.1 (28.6-100.6) |  | n.s. |
|  | CC | 157 | 35.7 (18.2–62.5) |  | 0.254^F^ | 69.0 (30.9-114.2) |  | n.s. | 66.6 (33.3-114.2) |  | n.s. |
| **rs4588*** | GG | 237 | 31.5 (17.0–60.6) | 0.096 | n.s. | 66.6 (30.3-111.9) | 0.394 | n.s. | 64.3 (29.8-105.9) | 0.434 | n.s. |
|  | GT | 188 | 27.7 (16.8–47.8) |  | n.s. | 61.9 (33.3-107.1) |  | n.s. | 59.5 (33.9-107.1) |  | n.s. |
|  | TT | 35 | 23.2 (16.3–36.4) |  | n.s. | 57.1 (26.2-80.3) |  | n.s. | 42.8 (25.0-89.3) |  | n.s. |
| **rs2282679*** | GG | 35 | 23.2 (16.3–36.4) | 0.085 | n.s. | 57.1 (26.2-80.3) | 0.418 | n.s. | 42.8 (25.0-89.3) | 0.434 | n.s. |
|  | TT | 236 | 31.5 (17.0–60.6) |  | n.s. | 66.6 (29.8-110.7) |  | n.s. | 64.3 (29.2-106.5) |  | n.s. |
|  | TG | 189 | 27.7 (16.7–47.6) |  | n.s. | 61.9 (33.3-107.7) |  | n.s. | 59.5 (33.5-107.1) |  | n.s. |
| **rs6013897*** | AA | 20 | 31.8 (14.8–53.0) | 0.916 | n.s. | 57.1 (32.1-66.6) | 0.211 | n.s. | 54.7 (32.1-76.2) | 0.833 | n.s. |
|  | TT | 294 | 29.2 (16.9–50.6) |  | n.s. | 65.5 (30.9-107.7) |  | n.s. | 61.9 (28.0-104.7) |  | n.s. |
|  | TA | 146 | 27.2 (17.0–52.6) |  | n.s. | 54.7 (28.6-108.3) |  | n.s. | 59.5 (33.3-111.9) |  | n.s. |
| **rs12785878** | GG | 43 | 24.1 (14.9–53.3) | 0.172 | n.s. | 66.6 (41.7-101.2) | 0.229 | n.s. | 59.5 (33.9-88.1) | 0.123 | n.s. |
|  | TT | 220 | 31.7 (18.0–56.2) |  | n.s. | 64.3 (33.3-111.9) |  | n.s. | 64.3 (35.7-111.9) |  | n.s. |
|  | TG | 198 | 26.1 (16.2–47.8) |  | n.s. | 59.5 (28.6-100.0) |  | n.s. | 52.4 (21.4-103.5) |  | n.s. |

*one is missing, ^A^= AA vs. GA, ^B^= GG vs. AA, ^C^=GA vs. GG, ^D^= AA vs. CA, ^E^= CA vs. CC, ^F^=CC v. AA, sample comparison was performed with the Dunn´s test ^59^

**Supplementary table 3:** **Distribution of SNP loci assessed regarding bacteraemia, infection and mortality rate**

| **SNP** | | **Bacteraemia** | | | **Infection** | | | **Survival** | | |
| --- | --- | --- | --- | --- | --- | --- | --- | --- | --- | --- |
|  |  | no (n=333) | yes (n=128) | p-value | no (n=135) | yes (n=326) | p-value | no (n=409) | yes (n=52) | p-value |
| **rs1993116*** | AA | 79 (69.3%) | 35 (30.7%) | 0.470 | 28 (24.6%) | 86 (75.4%) | 0.236 | 100 (87.7%) | 14 (12.3%) | 0.733 |
|  | GG | 115 (71.9%) | 45 (28.1%) | 0.913 | 46 (28.8%) | 114 (71.3%) | 0.915 | 143 (89.4%) | 17 (10.6%) | 0.877 |
|  | GA | 138 (74.2%) | 48 (25.8%) | 0.460 | 61 (32.8%) | 125 (67.2%) | 0.177 | 165 (88.7%) | 21 (11.3%) | >0.999 |
| **rs10741657** | AA | 52 (64.2%) | 29 (35.8%) | 0.078 | 19 (23.5%) | 62 (76.5%) | 0.228 | 69 (85.2%) | 12 (14.8%) | 0.252 |
|  | GG | 117 (71.8%) | 46 (28.2%) | 0.913 | 48 (29.3%) | 115 (70.6%) | >0.999 | 146 (89.6%) | 17 (10.4%) | 0.759 |
|  | GA | 164 (75.6%) | 53 (24.4%) | 0.145 | 68 (31.3%) | 149 (68.7%) | 0.412 | 194 (89.4%) | 23 (10.6%) | 0.768 |
| **rs7041*** | AA | 60 (80.0%) | 15 (20.0%) | 0.121 | 19 (25.3%) | 56 (74.7%) | 0.489 | 67 (89.3%) | 8 (10.7%) | >0.999 |
|  | CA | 156 (68.4%) | 72 (31.6%) | 0.077 | 64 (28.1%) | 164 (71.9%) | 0.609 | 201 (88.2%) | 27 (11.8%) | 0.769 |
|  | CC | 117 (74.5%) | 40 (25.5%) | 0.445 | 52 (33.1%) | 105 (66.9%) | 0.197 | 141 (89.8%) | 16 (10.2%) | 0.644 |
| **rs4588*** | GG | 168 (70.9%) | 69 (29.1%) | 0.533 | 72 (30.4%) | 165 (69.6%) | 0.610 | 211 (89.0%) | 26 (11.0%) | 0.883 |
|  | GT | 137 (72.9%) | 51 (27.1%) | 0.833 | 54 (28.7%) | 134 (71.3%) | 0.836 | 166 (88.3%) | 22 (11.7%) | 0.881 |
|  | TT | 28 (80.0%) | 7 (20.0%) | 0.332 | 9 (25.7%) | 26 (74.3%) | 0.703 | 32 (91.4%) | 3 (8.6%) | 0.784 |
| **rs2282679*** | GG | 28 (80.0%) | 7 (20.0%) | 0.332 | 9 (25.7%) | 26 (74.3%) | 0.703 | 32 (91.4%) | 3 (8.6%) | 0.784 |
|  | TT | 167 (70.8%) | 69 (29.2%) | 0.533 | 72 (30.5%) | 164 (69.5%) | 0.609 | 210 (89.0%) | 26 (11.0%) | 0.884 |
|  | TG | 138 (73.0%) | 51 (27.0%) | 0.833 | 54 (28.6%) | 135 (71.4%) | 0.835 | 167 (88.4%) | 22 (11.6%) | 0.881 |
| **rs6013897*** | AA | 15 (75.0%) | 5 (25.0%) | >0.999 | 6 (30.0%) | 14 (70.0%) | >0.999 | 15 (75.0%) | 5 (25.0%) | 0.062 |
|  | TT | 213 (72.4%) | 81 (27.6%) | 0.914 | 86 (29.3%) | 208 (70.7%) | >0.999 | 265 (90.1%) | 29 (9.9%) | 0.222 |
|  | TA | 105 (71.9%) | 41 (28.1%) | 0.911 | 43 (29.3%) | 103 (70.5%) | >0.999 | 129 (88.4%) | 17 (11.6%) | 0.875 |
| **rs12785878** | GG | 33 (76.7%) | 10 (23.3%) | 0.593 | 15 (34.9%) | 28 (65.1%) | 0.385 | 41 (95.3%) | 2 (4.7%) | 0.205 |
|  | TT | 157 (72.4%) | 63 (27.6%) | 0.755 | 59 (26.8%) | 161 (73.2%) | 0.306 | 188 (85.5%) | 32 (14.5%) | 0.039 |
|  | TG | 144 (72.2%) | 55 (27.8%) | >0.999 | 61 (30.8%) | 137 (69.2%) | 0.537 | 180 (90.9%) | 18 (9.1%) | 0.235 |

*one is missing, comparison was performed with the Mann-Whitney U test
